# Enhancing work environments and reducing turnover intention: a multicenter longitudinal cohort study on differentiated nursing practices in Dutch hospitals

**DOI:** 10.1186/s12912-024-02681-7

**Published:** 2025-01-10

**Authors:** Julia van Kraaij, Neeltje de Vries, Hanna Wessel, Hester Vermeulen, Catharina van Oostveen, RN2Blend Consortium, Julia van Kraaij, Hester Vermeulen, Catharina van Oostveen, Lisette Schoonhoven, Dewi Stalpers, Pieterbas Lalleman, Dieke Martini, Hugo Schalkwijk, Jet Spits, Roland Bal, Lucas Goossens, Iris Wallenburg, Martijn Felder, Syb Kuijper, Nienke Miedema

**Affiliations:** 1 Radboud University Medical Center, IQ Health Science Department, P.O. Box 9101, Nijmegen, 6525 EP The Netherlands; 2 Department of Internal Medicine, Spaarne Gasthuis Hospital, Hoofddorp, the Netherlands; 3 Spaarne Gasthuis Academy, Spaarne Gasthuis Hospital, Hoofddorp, the Netherlands; 4 Erasmus School of Health Policy & Management, Erasmus University Rotterdam, Rotterdam, The Netherlands; 5 HAN University of Applied Sciences, School of Health Studies, Nijmegen, The Netherlands

**Keywords:** Nursing work environment, Intention to leave, Turnover intention, Differentiated nursing practice, Nursing workforce, Multicenter longitudinal study

## Abstract

**Background:**

Addressing the growing challenge of nurse retention requires coordinated actions at national and global levels to improve recruitment, retention policies, and investments in the nursing work environment. The nursing work environment, defined as the "organizational characteristics of a work setting that facilitate or constrain professional nursing practice", is critical in influencing whether nurses decide to leave their jobs. This study investigates the impact of differentiated nursing practices – which involved tailoring roles and responsibilities based on nurses’ training, skills, and experience in Dutch hospitals – and investigated their impact on the nursing work environment and turnover intention (i.e., nurses’ intentions to leave their organization). We also explored whether the work environment mediates this relationship.

**Methods:**

A multicenter longitudinal cohort study was conducted across 19 Dutch hospitals between 2019 and 2023. Nursing professionals participated via digital surveys administered before (T0) and after (T1) differentiated nursing practices were introduced. The nursing work environment was assessed using the Practice Environment Scale of the Nursing Work Index. A multilevel analysis with a random intercept and fixed slope was used to evaluate the impact of differentiated nursing practices on the work environment and on nurses' turnover intentions.

**Results:**

We received 5411 responses to our questionnaire – 4259 at T0 and 1152 at T1. Results showed that, while the overall work environment improved, particularly in the dimensions of *staffing and resource adequacy*, *collegial nurse–physician relationships*, and *participation in hospital affairs*, there were no significant improvements in *nursing foundation for quality of care* or *nurse managers' ability, leadership, and support of nurses*. Additionally, differentiated nursing practices did not significantly impact turnover intention, nor did the work environment mediate this relationship.

**Conclusions:**

This study is the first to explore the unique effects of practice differentiation on turnover intention mediated by the work environment. The findings suggest that, while differentiated practices can enhance certain aspects of the work environment, a more systemic and integrated approach is required for sustained improvements. Future research should include longer term studies to fully understand the complex relationship and accompanying mechanisms between differentiated nursing practices, the nursing work environment, and turnover intention.

**Trial registration:**

Clinical trial number not applicable.

**Supplementary Information:**

The online version contains supplementary material available at 10.1186/s12912-024-02681-7.

## Background

Nurses represent over fifty percent of the health workforce and play a pivotal role in healthcare systems worldwide; however, anticipated staff shortages pose a significant challenge for the decades ahead [1]. High turnover rates among nurses have exacerbated this concern. For example, a recent literature review estimated a nurse turnover rate of 18% across countries in Europe, North America, and Asia [2]. Key risk factors for nurse turnover include being young and having lower education levels, low salaries, and heavy workloads. Dissatisfaction with the job and organization have also contributed to higher turnover rates [2]. Multiple studies have linked nurse staff turnover to rising healthcare costs and compromised quality and safety of patient care [3]. Effectively addressing this issue demands coordinated actions on national and global scales to establish recruitment and retention policies. Investment in the nursing work environment has also been identified as a way to reduce the number of nurses leaving their current jobs, or even the profession [4, 5].

The nursing work environment is defined as the “organizational characteristics of a work setting that facilitate or constrain professional nursing practice” ([6], p. 178), and plays a crucial role in turnover intention (i.e., the nurses’ intentions to leave their organization) [7]. Numerous factors in the nursing work environment affect retention, such as the organizational structure, culture, job demands, working conditions, staffing levels, and teamwork dynamics [4, 8]. For instance, Raso et al. (2021) found that direct care nurses were more likely to leave, suggesting a potential link between job roles and retention dynamics [9]. Moreover, providing development opportunities for nurses has been associated with reduced turnover intention [4]. These opportunities provide nurses with essential skills to navigate complex care demands and underscore organizational investment in their growth and wellbeing [10]. These findings show that hospitals can improve nurse retention by addressing various factors, preferably in combination, while fostering a stimulating work environment [4, 11].

Strategies to improve the nursing work environment vary across countries and even among organizations within the same country. This variation may be due to differences in systems, cultural norms, available resources, and organizational vision and leadership [12, 13]. It is therefore crucial to integrate the unique local context of hospital wards when tailoring intervention studies to evaluate nurse retention strategies [14]. While a significant body of nursing literature has provided strategies for improving nursing work environments, the long-term effectiveness of these interventions in retaining nurses has not been investigated [15, 16]. Longitudinal data are needed to determine the sustainability of retention strategies. This research gap may be limiting the ability of healthcare organizations to make informed decisions and effectively implement evidence-based practices to retain their nurses and create supportive work environments.

This longitudinal study aimed to explore the pivotal role of the nursing work environment in mitigating nurses' turnover intention and fostering a sustainable nursing workforce. Various initiatives have been introduced worldwide to enhance the appeal of the nursing profession by improving the nursing work environment with new roles and positions. For example, in the United Kingdom, nurse associates support healthcare teams by allowing registered nurses to handle more complex tasks thereby bridging the gap between registered nurses and healthcare assistants [17]. Similarly, in the United States, establishing clinical nurse leaders has improved patient outcomes using evidence-based practice to bridge the gap between administrative and clinical nurse roles [18]. In the Netherlands, hospitals have been adopting differentiated nursing practices. These practices involve tailoring roles and responsibilities based on nurses’ training, skills, and experience, enabling hospitals to optimize their nursing workforce. In addition, differentiated practices are supported by professional governance structures to promote work autonomy and strategically position nurses within their organizations. These practices aim to strengthen nursing professionalization and attract more individuals to the profession by expanding career development opportunities [13, 19].

Recent data from the Netherlands has indicated that 15% of nurses intended to leave the hospital, while 19.2% intended to leave the profession altogether [20]. This situation highlights the need for further exploration of differentiated nursing practices. Dutch nursing work environments are known for their complexity, numerous dependencies, and challenges in daily work processes [21]. Transitioning to differentiated nursing practices could strengthen organizational capacity and address factors contributing to the number of nurses leaving the profession [11, 13]. To investigate this further, we examined how differentiated nursing practices affected the nursing work environment and turnover intention. We also examined the relationships between these three factors.

## Methods

This study was designed and executed following the Strengthening the Reporting of Observational Studies in Epidemiology (STROBE) guidelines [22].

### Hypotheses

**Fig. 1:**
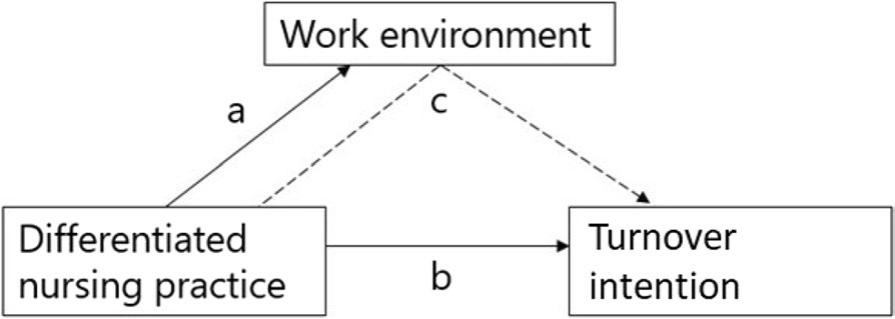
Conceptual analytical framework of this study

Based on our aim to explore the pivotal role of the nursing work environment in reducing turnover intention, we formulated three hypotheses (Fig. 1):

1. Differentiated nursing practices have a positive impact on the work environment (a)
2. Differentiated nursing practices reduce turnover intention among nurses (b)
3. The work environment mediates the relationship between differentiated nursing practice and turnover intention (c).

### Study design and setting

This multicenter longitudinal cohort study was conducted in nineteen Dutch hospitals, comprising six academic, nine teaching, and four general hospitals. The study is part of the nationwide Registered Nurses to Blend (RN2Blend) research program, which is examining differentiated nursing practice in the Netherlands [23].

### Intervention

The intervention involved introducing differentiated nursing practices, in which nursing roles and responsibilities were assigned based on each nurse’s education level, work experience, and expertise [13, 24, 25].

Differentiated practices focused on supported and facilitated professional governance by establishing structures that promote work autonomy and strategically positioning nurses within the organization through shared governance structures [13, 26]. Consequently, nurses were empowered to make decisions in clinical practice, to contribute to organizational strategy to support effective work practices, and to guide the strategy of these policies within structured governance systems. Moreover, the intervention engaged professional growth and leadership within the various nursing roles. By emphasizing leadership in decision-making, accountability, and ownership of responsibilities in the new nursing roles [27], the intervention sought to encourage nurses to take on more active roles within the healthcare environment [25]. Hence, nurses were provided with structured development opportunities to enhance their leadership skills and became more involved in strategic and quality-related decision-making.

In practice, the intervention’s core elements were enrolled by introducing the role of nurse coordinator alongside the existing nurse role [21, 25]. Some hospitals established these roles based on prior nursing education, creating and integrating differentiated positions for nurses trained at the vocational level and nurse coordinators with a bachelor’s degree. In contrast, other hospitals chose to focus less on educational background, while basing the roles on work experience, personal ambitions, and demonstrated competencies. Additionally, hospitals varied in how they defined and implemented the role of the nurse coordinator, leading to differences in responsibilities, and scope of practice [13].

Importantly, the intervention did not follow a strict national protocol; instead, each intervention was locally tailored, allowing hospitals to implement the differentiation flexibly and adapt it to the context of their organizations [13]. This adaptability allowed hospitals to design and implement the intervention in practice-based ways that best supported their organizational characteristics and goals [27]. Even within the same hospital, the application of the intervention varied across departments, as the specialty areas and operational needs differed. For a more comprehensive understanding, Additional File 1 presents a detailed description of a hospital intervention example, offering insights into the practical steps taken and adjustments made during the transition.

### Data collection procedures

The Nursing Quality Monitor (NQM) was used to collect data. This online questionnaire evaluates the quality of nursing care in Dutch hospitals, following Donabedian's structure-process-outcome quality-of-care model [28]. The NQM covers aspects related to nursing structure (e.g., educational and staffing levels), the process of nursing care (e.g., work environment and missed nursing care), and outcomes (e.g. nurses’ perceived quality of care and intention to leave). For this study, we focused on questions concerning the nursing work environment and turnover intention.

Data were collected and the NQM questionnaires distributed by Newcom, an independent research bureau in the Netherlands [29]. Newcom provided the survey link to collaborating hospitals or departments, who then forwarded it to eligible participants, inviting them to complete the NQM survey in Dutch. Completing the NQM survey took participants approximately 30 min. Surveys were administered at baseline (T0) and at least one year after the differentiated nursing practices were introduced (T1). The time interval between T0 and T1 varied depending on the hospital or department’s project planning.

### Participants

Aligned with the project planning for differentiated nursing practices within each specific hospital or department, all nursing professionals working in the participating wards were invited to complete the NQM survey through convenience sampling. The survey was also distributed among third- and fourth-year nursing students as they cover all nursing roles, including direct and indirect patient care activities and non-patient care-related nursing tasks [30]. These roles are typically accounted for in the ward's full-time equivalent budget [31].

For this study, we only used data collected from registered nurses working on clinical nursing wards. This excluded nurse assistants, first- and second-year nursing students, registered nurses in outpatient clinics, nurses in functional departments (such as the endoscopy ward), unit managers, and cluster managers from the study.

Participants were independently sampled at each measurement point (T0 and T1) through purposive sampling managed by each participating hospital, which determined the distribution of questionnaires across nursing wards. As a result, data were collected from different individuals at each time point, with no tracking of participants across T0 and T1.

### Measurements

#### Descriptive data

We collected sociodemographic variables, including gender (male, female), age, educational level (vocational training, diploma, specialized training, bachelor’s degree, master’s degree, PhD), full-time (≥ 32 h/week) or part-time (< 32 h/week) employment, years of experience in nursing, years of experience in the current hospital, ward type (medical, surgical, acute, mixed, and other) (see Table 1), and hospital type (general, teaching, academic).

**Table 1:** Ward types

**Medical ward:** Nurses care for patients who are treated with medication rather than surgical interventions. This includes internal medicine, psychiatry, and gastroenterology. **Surgical ward:** Nurses care for patients who are waiting for operative treatment or have already undergone an operation. **Acute ward:** Nurses care for patients requiring acute attention for severe injuries or critical episodes of illness. This includes the intensive care unit, coronary care unit, and emergency room. **Mixed ward:** Nurses care for patients with a combination of medical and/or surgical issues. Examples include gynecology and gastroenterological surgery. **Other ward:** Nurses work in roles that do not fit the above categories, such as in day care, dialysis, or as float nurses who work across various wards.	

#### Nursing work environment

A Dutch translation of the revised version of The Practice Environment Scale of Nursing Work Index (PES-NWI) was used to measure the nursing work environment [32]. The PES-NWI is an internationally validated instrument that includes 32 items, each with a four-point Likert scale comprising strongly disagree, disagree, agree, or strongly agree. The items are evaluated into five subscales of work environment: *staffing and resource adequacy* (four items), *collegial nurse–physician relationships* (seven items), *nurse manager ability, leadership, and support of nurses* (four items), *nurse participation in hospital affairs* (eight items), and *nursing foundation of quality of care* (nine items) [6]. The PES-NWI subscales and overall score (PES-NWI total) are represented as a composite measure by calculating the average of the relevant items (score varying from 1 to 4), and have high predictive validity for workforce stability issues in hospitals [33]. A score ≥ 2.5 is a positive assessment of the work environment [6]. The Dutch translation of the PES-NWI of the RN4CAST-consortium was used [32]. The reliability, in terms of Cronbach’s alpha, of this version of the PES-NWI subscales varies from 0.56 to 0.84 [33].

#### Turnover intention

Turnover intention was measured using the following dichotomous question: “If possible, would you leave the hospital within the next year because of dissatisfaction with your job?” This question was based on earlier research from RN4CAST [34], and is considered a good predictor of actual turnover [32, 35].

### Data preparation

For robust analysis, data were checked against specific criteria, and only hospitals with at least two participating wards were included in the analysis. Additionally, at least half plus one of the items ((n/2) + 1) needed to be completed for inclusion in the PES-NWI subscales. No respondents were excluded from the analysis based on these criteria.

### Data analysis

IBM SPSS Statistics 29 software (IBM Corp. IBM SPSS Statistics for Windows) was used for the analysis.

#### Descriptive analysis

Descriptive analyses are presented at T0 and T1 for participants’ sociodemographic characteristics, perceptions of their work environment, and turnover intention. Frequencies (n) and percentages (%) were calculated for categorical variables. Continuous normal-distributed data are presented as mean and standard deviation (SD). The Chi-square test and independent t-test were used to compare categorical and continuous variables between T0 and T1, respectively.

#### Multilevel analysis

While the nursing work environment is context bound [6], we assumed there would be differences between hospitals that could influence our result. Conventional regression analyses ignores these differences [36], so we performed a two-level multilevel model analysis (random intercept model) to account for clustering of nurses within hospitals. Moreover, given the cross-sectional sampling at T0 and T1, multilevel analysis is functional for clustering at the organizational level to examine patterns in intent to leave as they relate to hospital or department characteristics rather than tracking individual changes over time. Thus, level 1 data (individual nurse level) included gender, age, educational level, full-time or part-time employment, experience, and type of ward. Level 2 data represented the type of hospital (general, teaching, or academic).

The ICC values were 0.147 for PES-NWI and 0.007 for turnover intention. Although these low ICC values suggested multilevel analysis might not be necessary for measuring the intervention effect on the turnover intention, we still chose multilevel models for better estimates of both fixed and random effects [37].

Three multilevel models (two-level) with random intercept and fixed slope were used to study the outcomes. Model 1 examined the impact of differentiated nursing practice on the work environment using the PES-NWI subscales and the PES-NWI total score (hypothesis a; Fig. 1). Model 2 examined the impact of differentiated nursing practice on turnover intention (hypothesis b; Fig. 1). Model 3 studied the mediating effect of the work environment (PES-NWI total score) on the relationship between differentiated nursing practice and turnover intention (hypothesis c; Fig. 1).

Normal distribution was tested using graphical evaluations. The variance inflation factor was also calculated to test the multicollinearity assumption for each independent variable. Based on these findings, we excluded ‘age’ and ‘years of experience in nursing’ from the multilevel model.

To ensure stability and reliability of the estimated effects of the multilevel models, a sample size of at least 290 participants was required, based on the rule that there should be at least 10 participants per category per variable [38]. Results of the multilevel analysis were presented using estimates (β) and standard error (SE), 95% confidence interval (CI), and −2 log likelihood values.

## Results

**Fig. 2:**
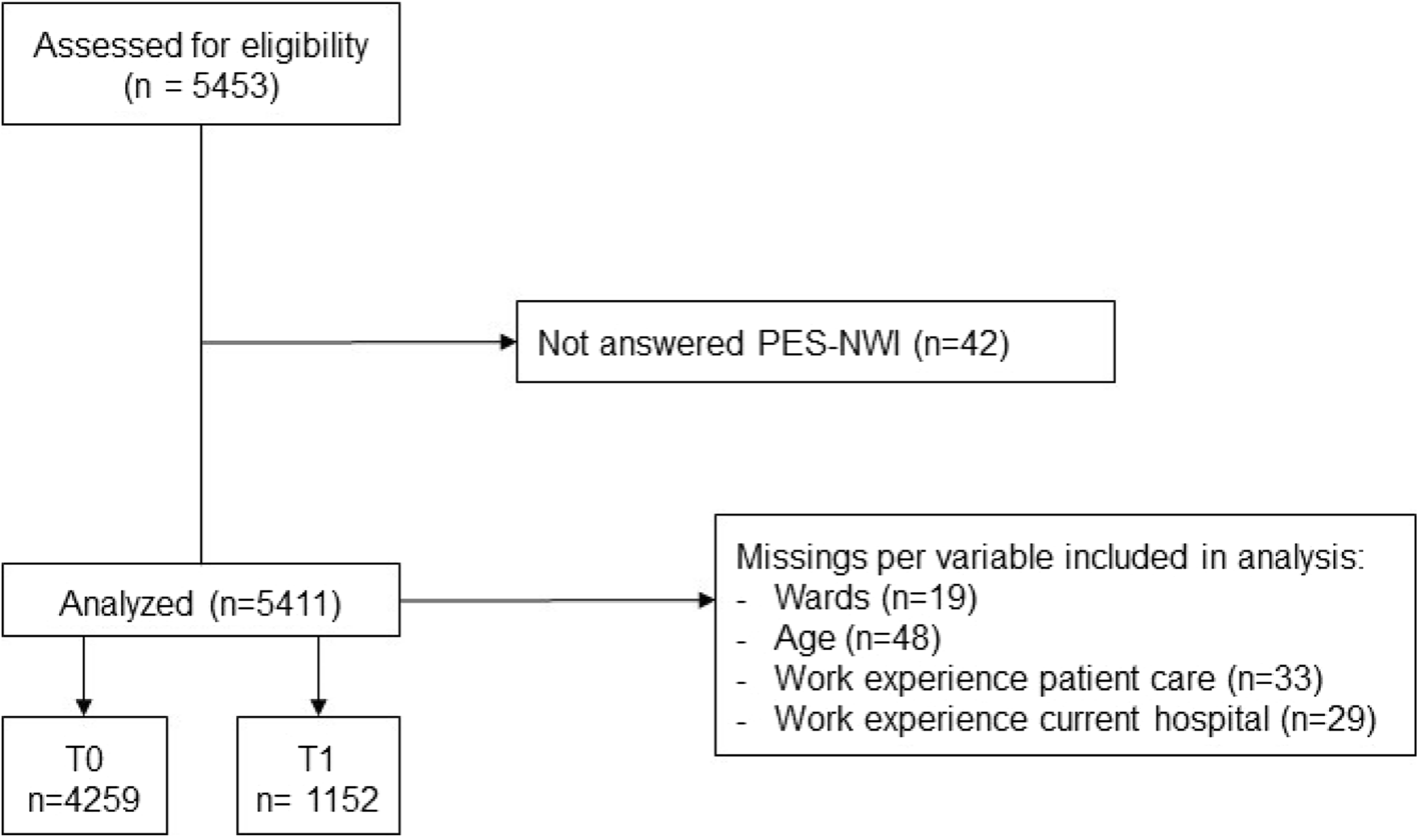
Study flowchart at T0 and T1 (TO = before differentiated nursing practices were introduced; T1 = after differentiated nursing practices were introduced)

We received completed questionnaires from 5411 nurses working in 19 hospitals throughout the Netherlands. We received 4259 of these before the differentiated nursing practices were introduced (T0) and 1152 afterwards (T1) (Fig. 2). The time between T0 and T1 varied from 7 months to three years.

### Descriptive data

The gender distribution and employment status were consistent at T0 and T1, with most nurses being female (88.4% at T0 and 88.2% at T1) and most being employed part-time (60.6% at T0 and 59.2% at T1) (Table 2). However, there were some differences between T0 and T1. The mean age of nurses was slightly lower at T1 (39.60 years, SD 12.77) than at T0 (40.62 years, SD 12.61). In addition, more nurses held a bachelor’s degree and a bachelor’s degree with specialized training at T1 than at T0 (25.6% and 25.7%, respectively at T1; 20.0% and 21.1%, respectively at T0). Additionally, nurses reported slightly less work experience in patient care at T1 (mean 17.02 years, SD 12.86) than at T0 (mean 18.13 years, SD 12.80) as well as less work experience in the current hospital at T1 (mean 13.21 years, SD 11.20) than at T0 (mean 14.02 years, SD 11.38).

**Table 2:** Sample characteristics and variables under study

	**T0** (*N* = *4259)*	**T1** *(N* = *1152)*	***P* value**
**Gender,** n (%)			0.881
Female	3763 (88.4)	1016 (88.2)	
Male	496 (11.6)	136 (11.8)	
**Age,** mean (SD)	40.62 (12.61)	39.60 (12.77)	0.016
**Education level,** n (%)			< 0.001
Vocational degree	595 (14.0)	134 (11.6)	
Vocational + specialized training	531 (12.5)	112 (9.7)	
Diploma	383 (9.0)	88 (7.6)	
Diploma + specialized training	914 (21.5)	201 (17.4)	
Bachelor’s degree	851 (20.0)	295 (25.6)	
Bachelor’s + specialized training	897 (21.1)	296 (25.7)	
Master’s degree	88 (2.1)	26 (2.3)	
**Employment,** n (%)			0.389
Full-time	1678 (39.4)	470 (40.8)	
Part-time	2581 (60.6)	682 (59.2)	
**Work experience in patient care (years),** mean (SD)	18.13 (12.80)	17.02 (12.86)	0.010
**Work experience current hospital (years),** mean (SD)	14.02 (11.38)	13.21 (11.20)	0.033
**Hospital type,** n (%)			< 0.001
Academic	1471 (34.5)	755 (65.5)	
Teaching	2349 (55.2)	340 (29.5)	
General	439 (10.3)	57 (4.9)	
**Ward**, n (%)			< 0.001
Medical	1367 (32.1)	368 (31.9)	
Surgical	954 (22.4)	368 (31.9)	
Acute	697 (16.4)	252 (21.9)	
Mixed	724 (17.0)	85 (7.4)	
Other	499 (11.7)	78 (6.8)	
**PES-NWI total,** mean (SD)	2.87 (0.38)	2.91 (0.36)	0.002
Staffing and resource adequacy	2.56 (0.60)	2.65 (0.57)	< 0.001
Collegial nurse–physician relationships	3.07 (0.48)	3.13 (0.47)	< 0.001
Nurse manager ability, leadership, and support of nurses	2.94 (0.61)	2.92 (0.62)	0.370
Nursing foundation of quality of care	2.97 (0.41)	2.97 (0.41)	0.807
Nurse participation in hospital affairs	2.69 (0.54)	2.76 (0.53)	< 0.001
**Turnover intention,** n (%)	1022 (24.0)	278 (24.1)	0.924

*N* population size, *PES-NWI* Practice Environment Scale of Nursing Work Index, *SD* standard deviation, *TO* digital surveys administered before differentiated nursing practices were introduced, *T1* digital surveys administered after differentiated nursing practices were introduced

We received responses from nurses working in various types of hospitals and wards. At T0, most participants were working in teaching hospitals (55.2%), whereas most were employed in academic hospitals at T1 (65.5%). There were also differences in the hospital wards nurses were working on between T0 and T1; medical and surgical wards were predominant types at both time points, but the proportions varied (Table 2).

There were modest differences in the total PES-NWI score and three of its subscales between T0 and T1. Overall, nurses perceived their work environment more favorably at T1 (mean score 2.91, SD 0.36) than at T0 (mean score 2.87, SD 0.38). Specifically, perceptions of *staffing and resource adequacy* increased from 2.56 (SD 0.60) at T0 to 2.65 (SD 0.57) at T1, *collegial nurse–physician relationships* improved from 3.07 (SD 0.48) to 3.13 (SD 0.47), and *nurse participation in hospital affairs* rose from 2.69 (SD 0.54) to 2.76 (SD 0.53). Turnover intention remained consistent, with rates of 24.0% at T0 and 24.1% at T1.

### Effect of differentiated practice on the nursing work environment

Introducing differentiated practices had a small effect on nurses’ perception of their work environment (Table 3; β = 0.054, 95% CI 0.027 to 0.081). We corrected this association for gender, age, education level, type of employment, work experience in the current hospital, and the type of hospital and ward. Although the type of hospital did not influence the association between differentiated practices and nurses’ perception of their work environment, nurses working in medical wards (β = − 0.056, 95% CI − 0.090 to − 0.022), surgical wards (β = − 0.086, 95% CI − 0.112 to − 0.050), acute wards (β = − 0.049, 95% CI − 0.087 to − 0.011), and mixed wards (β = − 0.075, 95% CI − 0.144 to − 0.011) perceived their work environment as less favorable than nurses working in other wards did. We also found that nurses with more work experience were more critical of their work environment (β = − 0.001, 95% CI − 0.002 to 0.000).

**Table 3:** Multilevel model effect differentiated practice on PES-NWI total

	**Estimate (β)**	**SE**	**95% CI**	***P* value**
**Intercept**	2.821	0.086	2.645 to 2.997*	
**Intervention**	0.054	0.014	0.027 to 0.081*	
**Gender**				0.465
Male	− 0.012	0.016	− 0.042 to 0.019	
Female	0	0		
**Education level**				0.001
Vocational degree	0.044	0.036	− 0.027 to 0.115	
Vocational + specialized training	0.028	0.036	− 0.043 to 0.099	
Diploma	− 0.042	0.039	− 0.116 to 0.032	
Diploma + specialized training	0.015	0.036	− 0.055 to 0.085	
Bachelor’s degree	0.052	0.035	− 0.016 to 0.121	
Bachelor’s degree + specialized training	0.022	0.035	− 0.046 to 0.090	
Master’s degree	0	0		
**Employment**				0.956
Part-time	− 0.001	0.011	− 0.022 to 0.020	
Full-time	0	0		
**Work experience current hospital (years)**	− 0.001	0.001	− 0.002 to 0.000*	
**Ward**				< 0.001
Medical	− 0.056	0.017	− 0.090 to − 0.022*	
Surgical	− 0.086	0.018	− 0.122 to − 0.050*	
Acute	− 0.049	0.019	− 0.087 to − 0.011*	
Mixed	− 0.075	0.020	− 0.114 to − 0.036*	
Other	0	0	0	
**Hospital type**				0.505
Academic	0.040	0.098	− 0.167 to 0.248	
Teaching	0.102	0.092	− 0.092 to 0.296	
General	0	0		
**Information criteria**				
** −2 log likelihood**	4170.118			

*SE* standard error, *CI* confidence interval

^*^P < 0.05

#### PES-NWI subscales

Differentiated practices had small positive effects on nurses’ perceptions of various subscales of their work environment (Table 4), including *staffing and resource adequacy* (β = 0.054, 95% CI 0.09 to 0.098), *collegial nurse–physician relationships* (β = 0.065, 95% CI 0.030 to 0.101), and *nurse participation in hospital affairs* (β = 0.099, 95% CI 0.060 to 0.139).

**Table 4:** Multilevel model impact on PES-NWI subscales

	**Subscale 1: Staffing and resource adequacy**	**Subscale 2: Collegial nurse–physician relationships**	**Subscale 3; Nurse manager ability, leadership, and support of nurses**	**Subscale 4: Nursing foundation of quality of care**	**Subscale 5: Nurse participation in hospital affairs**
	*β (95% CI)*	*β (95% CI)*	*β (95% CI)*	*β (95% CI)*	*β (95% CI)*
**Intercept**	2.652 (2.434 to 2.869*)	2.950 (2.782 to 3.117*)	2.986 (2.723 to 3.250*)	2.856 (2.686 to 3.027*)	2.704 (2.474 to 2.934*)
**Intervention**	0.054 (0.009 to 0.098*)	0.065 (0.030 to 0.101*)	0.007 (− 0.038 to 0.053)	0.026 (− 0.005 to 0.056)	0.099 (0.060 to 0.139*)
**Gender**					
Male	0.049 (− 0.001 to 0.099)	− 0.005 (− 0.045 to 0.036)	− 0.051 (− 0.103 to 0.000)	− 0.017 (− 0.052 to 0.018)	− 0.022 (− 0.066 to 0.023)
Female	0	0	0	0	0
**Education level**					
Vocational degree	− 0.096 (− 0.211 to 0.020)	0.027 (− 0.066 to 0.120)	− 0.018 (− 0.136 to 0.100)	0.103 (0.023 to 0.182*)	0.090 (− 0.012 to 0.193)
Vocational + specialized training	− 0.068 (− 0.184 to 0.048)	0.080 (− 0.013 to 0.173)	− 0.085 (− 0.203 to 0.033)	0.094 (0.015 to 0.174*)	0.009 (− 0.094 to 0.112)
Diploma	− 0.148 (− 0.268 to − 0.028)	− 0.057 (− 0.154 to 0.040)	− 0.136 (− 0.259 to − 0.013*)	0.029 (− 0.054 to 0.112)	− 0.017 (− 0.124 to 0.090)
Diploma + specialized training	− 0.087 (− 0.200 to 0.026)	0.087 (− 0.005 to 0.178)	− 0.077 (− 0.193 to 0.039)	0.073 (− 0.005 to 0.151)	− 0.020 (− 0.121 to 0.081)
Bachelor’s degree	− 0.039 (− 0.151 to 0.073)	0.055 (− 0.035 to 0.146)	0.018 (− 0.097 to 0.132)	0.089 (0.012 to 0.166*)	0.067 (− 0.032 to 0.167)
Bachelor’s degree + specialized training	− 0.044 (− 0.155 to 0.067)	0.104 (0.015 to 0.194*)	− 0.046 (− 0.159 to 0.068)	0.057 (− 0.020 to 0.134)	− 0.026 (− 0.125 to 0.073)
Master’s degree	0	0	0	0	0
**Employment**					
Part-time	− 0.014 (− 0.048 to 0.021)	0.007 (− 0.020 to 0.035)	0.015 (− 0.019 to 0.050)	− 0.007 (− 0.031 to 0.016)	− 0.001 (− 0.032 to 0.029)
Full-time	0	0	0	0	0
**Work experience current hospital (years)**	− 0.001 (− 0.003 to 0.001)	− 0.001 (− 0.002 to 0.00)	− 0.003 (− 0.005 to − 0.001*)	0.000 (− 0.001 to 0.002)	− 0.003 (− 0.004 to − 0.001*)
**Ward**					
Medical	− 0.170 (− 0.226 to − 0.115*)	0.023 (− 0.016 to 0.074)	0.004 (− 0.053 to 0.061)	− 0.122 (− 0.160 to − 0.084*)	− 0.030 (− 0.080 to 0.019)
Surgical	− 0.144 (− 0.202 to − 0.086*)	0.024 (− 0.070 to 0.025)	− 0.028 (− 0.088 to 0.031)	− 0.128 (− 0.169 to 0.088*)	− 0.098 (− 0.150 to − 0.046*)
Acute	− 0.009 (− 0.071 to 0.053)	0.025 (0.002 to 0.102*)	− 0.030 (− 0.093 to 0.033)	− 0.105 (0.148 to − 0.063*)	− 0.105 (− 0.160 to − 0.050*)
Mixed	− 0.182 (− 0.245 to − 0.119)	0.026 (0.003 to 0.105*)	− 0.051 (− 0.166 to 0.013)	− 0.145 (− 0.189 to − 0.102*)	− 0.069 (− 0.125 to − 0.012*)
Other	0	0	0	0	0
**Hospital type**					
Academic	0.122 (− 0.114 to 0.359)	0.043 (− 0.136 to 0.221)	− 0.039 (− 0.342 to 0.265)	0.083 (− 0.111 to 0.278)	− 0.038 (− 0.304 to 0.227)
Teaching	0.071 (− 0.150 to 0.292)	0.012 (− 0.155 to 0.178)	0.102 (− 0.182 to 0.385)	0.157 (− 0.025 to 0.338)	0.108 (− 0.139 to 0.356)
General	0	0	0	0	0
**−2 log likelihood**	9344.417	7054.674	9590.346	5399.985	8120.049

*β* estimate, *CI* confidence interval

^*^P < 0.05

The analyses showed that diploma nurses perceived slightly more support from their managers than those with other educational backgrounds did (β = − 0.136, 95% CI − 0.259 to − 0.013), and that nurses with a bachelor’s degree and specialized training perceived better collegial relationships (β = 0.104, 95% CI 0.0105 to 0.194). Nurses with a vocational degree and specialized training and those with a bachelor’s degree reported more positively on the foundational elements necessary to deliver high-quality of care. More experienced nurses reported less support from nurse managers (β = − 0.003, 95% CI − 0.005 to − 0.001) and felt less involved in hospital affairs (β = − 0.003, 95% CI − 0.004 to − 0.001). Nurses’ perceptions of their work environment also varied based on the ward they worked in. Those in medical wards (β = − 0.170, 95% CI − 0.226 to − 0.115) and surgical wards (β = − 0.144, 95% CI − 0.202 to − 0.086) reported less staffing and resource adequacy, while nurses working in acute (β = 0.025, 95% CI 0.002 to 0.102) and mixed (β = 0.026, 95% CI 0.003 to 0.105) wards reported better collegial relationships. Nurses working in surgical wards also perceived a lower quality of care (β = − 0.128, 95% CI − 0.169 to 0.088) as well as less participation in hospital affairs (β = − 0.098, 95% CI − 0.150 to − 0.046). Nurses working in all wards perceived a lower quality of care than those working on other wards (medical: β = − 0.122, 95% CI − 0.160 to − 0.084; surgical: β = − 0.128, 95% CI − 0.169 to 0.088; acute: β = − 0.105, 95% CI − 0.148 to − 0.063; mixed: β = − 0.145, 95% CI − 0.189 to − 0.102). Lastly, nurses working in surgical (β = − 0.098, 95% CI − 0.150 to − 0.046), acute (β = − 0.105, 95% CI − 0.160 to − 0.050), and mixed (β = − 0.069, 95% CI − 0.125 to − 0.012) wards experienced less participation in hospital affairs.

### Effect of differentiated practice on turnover intention

The introduction of differentiated practice did not affect turnover intention (β = − 0.051, SE = 0.087, 95% CI − 0.221 to 0.119).

### Effect of differentiated practice on turnover intention via the nursing work environment

The nursing work environment had no effect on turnover intention (β = 0.069, 95% CI − 0.113 to 0.252), suggesting the work environment does not mediate the impact of differentiated practice on turnover intention.

## Discussion

### Main findings

This study explored the crucial role of the nursing work environment in reducing turnover intention in Dutch hospitals through differentiated nursing practice. Our first hypothesis was that differentiated practice would positively impact nurses' perception of their work environment. The results supported this hypothesis, showing an overall improvement in the perceived work environment, particularly in the subscales *staffing and resource adequacy, collegial nurse–physician relationships*, and *participation in hospital affairs*. However, there was no significant improvement in *nursing foundation for quality of care* and *nurse managers' ability, leadership, and support of nurses.* A previous Dutch study by Bloemhof et al. (2021) evaluated a program that aimed to improve the professional work environment, enhance nurses' expertise, and elevate nurses’ roles [39]. This evaluation was based on the measurements of the Essentials of Magnetism and showed improvements in seven areas, except for adequacy of staffing. This is in contrast to our findings, which showed an improvement in staffing adequacy after the introduction of our differentiated nursing practice. However, comparing these results is challenging because of differences in study design. Bloemhof et al. assessed a specific hospital intervention, while we evaluated the general principles of practice differentiation. Additionally, the Essentials of Magnetism used by Bloemhof et al. and the PES-NWI used in our study measure different aspects of the work environment.

Our results did not support our second hypothesis that differentiated nursing practice would reduce nurses' turnover intention. Similarly, our third hypothesis that the work environment would mediate this relationship was also not supported. We did observe slight improvements in three of the five PES NWI subscales, i.e. *staffing and resource adequacy, collegial nurse–physician relationships*, and *participation in hospital affairs*, but this did not affect turnover intention. This contrasts with earlier research linking a better work environment with decreased turnover intention [7, 40]. An earlier study conducted across ten countries, including the Netherlands, found that three subscales were significantly linked to turnover intention among nurses. These subscales were *nurse managers' ability, leadership, and support of nurses*, *collegial nurse–physician relationships*, and *participation in hospital affairs* [41].

Differentiated nursing practices are context-specific and vary across organizations, making them difficult to standardize and define [13, 42]. However, they typically involve creating new roles and providing supportive structures for professional governance [13]. These mechanisms are often linked to strategies to improve the nursing work environment [43–46]. The first mechanism of differentiated practice involves creating new, future-oriented nursing roles, with hospitals increasingly differentiating nurses based on their competencies and education levels. For instance, many hospitals have introduced nurse coordinator positions to manage patient care, improve quality of care, and support other nurses [25]. These positions often require nurses with bachelor's degrees or bachelor competencies. Increasing the proportion of bachelor-trained nurses and aligning their roles with their competencies may enhance the work environment. This is supported by research indicating that a higher skill mix of bachelor-trained nurses is associated with better work environments and improved outcomes for both patients and nurses [47].

The introduction of differentiated practices generally improved perceived staffing and resource adequacy, but not for nurses working on medical and surgical wards. A potential explanation is that practice differentiation in medical and surgical wards may have led to bachelor-educated nurses balancing direct patient care with additional responsibilities, leading to insufficient registered nurses to manage the workload and maintain quality of care [47]. Van der Mark et al. confirmed this theory, noting that nurses reported difficulty in completing quality and organizational tasks, such as adhering to protocols and guiding students, when assessing staffing adequacy [48]. These findings underscore the need for a long-term vision on differentiated practices that actively involves all nurses.

The second mechanism of differentiated nursing practice focuses on promoting professional nursing governance through differentiated nursing roles. This approach emphasizes nurses’ accountability, ownership, and decision-making in clinical practice. We found that this approach improved *collegial nurse–physician relationships* and *nurse participation in hospital affairs* (albeit slightly). These findings align with those of previous research showing that enhancing collaboration between nurses and physicians and increasing nurse autonomy requires a multifaceted approach, including active participation of nurses in decision-making [49, 50]. However, we did not observe improvements in the *nursing foundation for quality of care* or in *nurse managers' ability, leadership, and support of nurses* after introducing differentiated nursing practices*.* Several factors may explain this. Increasing opportunities for nurses to engage in decision-making may enhance their perception of involvement, even if the foundational aspects of care or managerial support have not yet improved. Experienced nurses, with higher expectations based on their practice, might be more critical of the work environment [51]. Furthermore, differentiated nursing practices, such as distinguishing between vocational and bachelor-trained nurses, have raised concerns among vocational nurses about role degradation [52]. More qualified nurses often assess the work environment and care quality more critically [53]. In addition, differentiated practices may target nurse participation in hospital affairs rather than addressing broader aspects like quality of care or managerial support. Effective managerial support and positive leadership are crucial for improving the nursing work environment.

Despite various interventions aimed at improving professional nursing governance, isolated approaches often fail to significantly improve the work environment [43]. The nursing work environment is complex and influenced by numerous factors, suggesting that isolated interventions may not fully address underlying issues [21]. Introducing differentiated practices is a complex change that cannot be implemented linearly [13]. Meaningful progress in the work environment requires integrated and transformational approaches, as changes in one part of the system will inevitably affect other parts [54]. Our findings indicate that differentiated practices can enhance collaboration and decision-making, contributing to a better work environment.

Inconsistent application of differentiated practices across hospitals may explain why these changes have not significantly reduced turnover intention. Bloemhof et al. (2021) have emphasized the benefits of a comprehensive model [39], while van Kraaij et al. (2024) have advocated for a systemic approach to transforming the work environment [21].

### Strengths and limitations

A strength of this study is that it is, to the best of our knowledge, the first to investigate the unique impact of practice differentiation on turnover intention among nurses, and how this is mediated by the nursing work environment. This study provides valuable insights for organizations that differentiate nursing practices. Another strength is the large sample size, which enhances the power and reliability of our findings [55].

There are also some limitations that need to be acknowledged. The first limitation is the absence of a standardized measure or protocol for practice differentiation, which means that each hospital or ward implemented differentiated practices in a slightly different manner. This could have affected the effect of the intervention. However, given the wide variety of tasks involved in nursing, which can differ significantly across patients, wards, and hospitals, differentiated practices must be adapted to specific contexts to ensure their appropriateness and effectiveness [13, 42]. Hence, it is important to avoid overly rigid protocols. The flexibility and customization within each ward or hospital play a vital role in successful implementation of differentiated practices [19].

The second limitation is that we included only one pre- and one post-intervention measurement, which may not have fully captured the effect of the intervention or the effect of other events during the measurement period. Differentiated nursing practices are introduced in phases in a non-linear process [13, 56]. Integrating this complex system, which involves multiple stakeholders, can take several years [57]. Nevertheless, our finding that the intervention influenced specific aspects of the nursing work environment is encouraging. Future research should use repeated measurements over time, such as a time-series analysis, to better understand the long-term effects of practice differentiation on the work environment, retention rates, and quality of patient care.

Lastly, the absence of data on response rates is a limitation, potentially introducing non-response bias. Nonetheless, the sample characteristics at T0 and T1 are closely aligned. This suggests that the findings are likely representative.

### Implications for practice and research

Our results highlight the importance of practice differentiation in improving the nursing work environment specifically with regard to *staffing and resource adequacy, collegial nurse–physician relationships*, and *participation in hospital affairs*. These findings highlight the role of differentiated practice in empowering nurses to actively shape and influence their work environment. However, not all components of the work environment were improved by practice differentiation and nearly a quarter of nurses are still considering leaving their organization. This indicates that not all aspects of the work environment are adequately addressed by the intervention. By considering the work environment as a system and influencing it as such, for example with comprehensive multicomponent programs as described by Bloemhof et al. [39], complex changes like practice differentiation can be implemented successfully. A clear vision of what nursing is and which positions belong to it and an involvement of nurses in shaping changes are important for this [13]. Managers can facilitate this by encouraging nurses to get involved in change processes, fostering positive collateral relationships, acknowledging nurse responsibility, investing in competency development for change, and creating a supportive environment where nurses feel valued, respected, and empowered to voice their concerns and ideas [58, 59]. Managers, hospital boards, and nursing associations should lead these initiatives, ensuring that input is incorporated from nurses at *all* levels. Collaborating with professional networks can further strengthen this approach, allowing for a more comprehensive and inclusive strategy. Fostering nurse ownership and encouraging participation can help create a more supportive and effective work environment [21].

Future research should investigate the complex relationship between differentiated nursing practices and turnover intention among nurses. While some aspects of the work environment were improved by differentiated practice, these improvements did not significantly reduce turnover intention, indicating a need for a deeper examination of how specific work environment factors influence retention and understand how these interact with differentiated practices. Longitudinal research is needed to assess the long-term impact of differentiated practices on nurse retention. By clarifying the mechanisms through which these practices affect the work environment, more targeted and effective interventions can be developed. A systems thinking research approach could provide a comprehensive view of how differentiated nursing practices influence the work environment [60].

## Conclusions

Differentiating nursing practices can improve the nursing work environment, especially staffing adequacy, nurse–physician relationships, and participation in hospital affairs. These findings underscore the value of practice differentiation in enabling nurses to influence and shape their work environment. This also shows hospital managers that differentiated nursing practice can create work environments that are more favorable to nurses and may encourage them to stay with the organization. However, to significantly increase nurse retention, a systemic and multifaceted approach to improving the nursing work environment may be required. Researchers can help achieve this using a longitudinal and systemic approach to improve our understanding of the mechanisms of and complex relationships between differentiated nursing practices, the nursing work environment, and turnover intention.

## Supplementary Information

Supplementary Material 1.

## Data Availability

The datasets generated and analyzed during this study are not publicly available because of privacy and confidentiality concerns but are available from the corresponding author on reasonable request.

## Abbreviations

STROBE: Strengthening the Reporting of Observational Studies in Epidemiology
RN2Blend: Registered Nurses to Blend
NQM: Nursing Quality Monitor
T0: Baseline measurement (before differentiated practices were introduced)
T1: Follow-up measurement (after differentiated practices were introduced)
SD: Standard deviation
PES-NWI: Practice Environment Scale of Nursing Work Index
CI: Confidence interval
SE: Standard error
ICC: Intra-class correlation coefficient
