# Supplementary material for: Enhancing work environments and reducing turnover intention: a multicenter longitudinal cohort study on differentiated nursing practices in Dutch hospitals
Source: BMC Nurs. 2025 Jan 10;24:39. doi: 10.1186/s12912-024-02681-7 (PMC11721496; doi:10.1186/s12912-024-02681-7)
Supplement: Supplementary file 1 — Supplementary Material 1. [file 12912_2024_2681_MOESM1_ESM.docx]

# **Additional file 1. Detailed description on the intervention in differentiated practices of one participating hospital**

Before the transition to differentiated nursing practices, nursing teams in hospital A consisted of vocationally and bachelor trained registered nurses. Despite existing educational differences between these registered nurses, they performed the same tasks and roles.

With the introduction of differentiated nursing practices, hospital A transitioned from a single nursing role to a more specialized structure, creating two distinct roles: **nurse** and **nurse coordinator** (Box 1). These roles align with vocational and bachelor-level training, respectively. This differentiation aimed to enhance patient care and improve team efficiency by aligning roles with the nurses’ educational backgrounds and competencies. As a result, vocationally trained nurses focus on hands-on, direct patient care, while bachelor-trained nurses, as nurse coordinators, are responsible for clinical leadership, complex clinical reasoning and decision-making, and promoting evidence-based practices.

**Box 1.** New roles in the differentiated structure

**Nurse**

Nurses in this role focus primarily on direct, hands-on patient care, including administering medications, assisting with daily needs, monitoring patient conditions, and communicating with other healthcare providers. This role may also involve participation in specific focus areas or departmental working groups, contributing to ongoing improvement initiatives within the department.

**Nurse coordinator**

Nurse coordinators have a broader scope of practice, balancing direct patient care with leadership and organizational responsibilities. They play a key role in guiding clinical and departmental processes at both the unit and hospital levels, as well as within broader care networks. Nurse coordinators are central to clinical reasoning and decision-making, particularly in complex cases, and work collaboratively with multidisciplinary teams. They also promote evidence-based practices, assess care outcomes, identify areas for improvement, and contribute to the development and revision of protocols and procedures based on current research. Nurse coordinators are responsible for coordinating department initiatives, and promoting the collaboration for the initiatives across organizational level.

Moreover, hospital A focused with the introduction of differentiated practices on the strategic positioning of nurses, especially those in the nurse coordinator role, to enhance their impact on patient care and organizational outcomes. This approach assigns nurse coordinators responsibilities that extend beyond patient care to include leadership and coordination of department-wide initiatives. Nurse coordinators translate national healthcare developments into departmental proposals, foster alignment in hospital-wide care delivery through standardized pathways, and lead (clinical) focus areas, i.e. complex clinical reasoning and evidence based practice for specific patient groups. They are accountable for planning, executing, and tracking these initiatives, coordinating with department heads and other units as needed. This strategic positioning reinforces their role as departmental leaders, empowering them to shape policies and practices aligned with patient needs and the hospital's objectives.

In alignment with the new differentiated structure hospital A also introduces distinct salary scales, with nurse coordinators positioned in a higher scale, providing them with greater potential for salary growth and career advancement. While nurse coordinators take a lead role in clinical decision-making, hospital A emphasizes a collaborative complementary approach, ensuring that both vocationally and bachelor-trained nurses contribute their unique skills to provide coordinated, holistic patient care.

Career development opportunities

During the transition, hospital A introduced new career pathways and educational opportunities for nursing staff. Vocationally trained nurses who wished to advance could complete bridge programs to obtain a bachelor’s degree, allowing them to transition to the nurse coordinator role. Nurse coordinators may receive additional training or undergo assessments to ensure they meet consistent competency levels across the role.

Transition period

The transition period for introducing differentiated nursing practices spanned three to five years, allowing departments time to adopt the new nurse and nurse coordinator roles fully. Initial steps involved assessing each department’s staffing needs and skill levels, followed by targeted training and development programs to support nurses transitioning to the new roles. Temporary additional staffing was allocated to ensure consistent patient care while departments adjusted to new roles and responsibilities.

During this transition period, the practical application of differentiated roles was tailored at the departmental level. Each department customized the differentiation according to its unique operational needs and areas of clinical specialization. For example, highly specialized departments with a demand for complex clinical reasoning prioritized the nurse coordinators' role in guiding challenging clinical case assessments and decision-making. In contrast, other departments focusing on the embedding of evidence-based practices designated nurse coordinators to supervise journal clubs and write critical appraised topics and protocols.

Management played an active role in the transition, and department heads facilitated open communication with staff. By the end of the transition, departments were expected to have fully integrated the new roles into their standard operations, with the nurse and nurse coordinator roles clearly defined and established within the teams.
